# Differences in Growth Properties among Two Human Cytomegalovirus Glycoprotein O Genotypes

**DOI:** 10.3389/fmicb.2017.01609

**Published:** 2017-08-22

**Authors:** Julia Kalser, Barbara Adler, Michael Mach, Barbara Kropff, Elisabeth Puchhammer-Stöckl, Irene Görzer

**Affiliations:** ^1^Center for Virology, Medical University of Vienna Vienna, Austria; ^2^Max von Pettenkofer-Institute for Virology, Ludwig-Maximilians-University Munich Munich, Germany; ^3^Institute of Clinical and Molecular Virology, Friedrich-Alexander University Erlangen-Nürnberg Erlangen, Germany

**Keywords:** HCMV, glycoprotein O, trimeric complex, tropism, epithelial cells, fibroblasts

## Abstract

Glycoprotein O (gO) of the human cytomegalovirus (HCMV) is the critical subunit of the envelope trimer gH/gL/gO as it interacts with platelet-derived growth factor alpha receptor upon fibroblast entry, and triggers gB-mediated fusion for fibroblast and epithelial cell infection. Eight genotypes (GT) of the highly polymorphic gO gene are described, yet it is unclear whether the distinct GTs differ in their function. Thus, we aimed to elucidate potential functional differences between two highly diverse gO GTs in an otherwise genomically identical HCMV strain. Therefore, resident gO GT1c sequence of strain TB40-BAC4-luc was entirely replaced by gO GT4 of strain Towne and both, GT1c and GT4 viruses, were investigated for their growth properties in fibroblasts and epithelial cells. In addition, two conserved gO cysteines involved in gH/gL/gO stabilization were mutated to serine either in GT1c (C218S and C343S) or GT4 (C216S and C336S) and their effects on cell-free infectivity were assessed. GT4 viruses displayed a significantly enhanced epithelial cell tropism and this resulted in higher virus release upon replication in epithelial cells when compared to GT1c viruses. Further, when the two cysteines were individually mutated in gO GT1c no impairment in cell-free infectivity was observed. This, however, was in sharp contrast to gO GT4, in which both of the corresponding cysteine mutations led to a substantial reduction in cell-free infectivity which was even more pronounced upon mutation of GT4-C336 than of GT4-C216. In conclusion, these findings provide evidence that the two highly diverse gO genotypes, GT1c and GT4, differ in their functional properties as revealed by their different infection capacities for epithelial cells and by their different responsiveness to mutation of strictly conserved cysteine residues. Thus, it is likely that the gO heterogeneity influences cell-free infectivity of HCMV also *in vivo* which may have important implications for virus host transmission.

## Introduction

Human cytomegalovirus (HCMV) is a ubiquitously distributed human betaherpesvirus with 40–90% of adults infected worldwide. HCMV infection in immunocompetent individuals is usually clinically silent, but may lead to severe clinical manifestations in congenitally infected newborns and is one of the most severe opportunistic viral infections in patients with a weakened immune system (Britt, 2008; Mocarski, 2013).

Human cytomegalovirus has a large dsDNA genome with about 236 kb coding for ∼165 genes (Dolan et al., 2004; Sijmons et al., 2014). Comprehensive sequence analysis of numerous HCMV strains showed that a subset of genomic regions, which often encode for genes involved in immunomodulation, entry, spread, and cell tropism, is particularly diverse (Dunn et al., 2003; Stern-Ginossar et al., 2012; Van Damme and Van Loock, 2014). Remarkably, for most of the polymorphic genes a limited number of genotypes can be defined and it appears that extensive recombinations between HCMV strains have led to a large variety of circulating HCMV strains differing in their genotypic combinations (Dolan et al., 2004; Puchhammer-Stöckl and Görzer, 2011; Sijmons et al., 2015).

Human cytomegalovirus infects a broad range of cell types *in vivo*, with epithelial cells, endothelial cells, fibroblasts and smooth muscle cells being the predominant targets for virus replication (Sinzger et al., 2008; Mocarski, 2013). Two envelope glycoprotein complexes, the trimer gH/gL/gO and the pentamer gH/gL/UL128L, seem to play an important role in cell tropism (Ryckman et al., 2010; Wille et al., 2010; Scrivano et al., 2011). Since both, gO and UL128, form a covalent bond with gL-C144 of the gH/gL heterodimer, the two complexes are mutually exclusive (Ciferri et al., 2015; Zhou et al., 2015). The current model proposes that the trimer is necessary for entry in all cell types while the pentamer is additionally required for entry into epithelial and endothelial cells (Jiang et al., 2008; Wille et al., 2010; Kabanova et al., 2016), and it is further suggested that the relative amounts of both complexes define the cell tropism of virus progenies (Murrell et al., 2013; Zhou et al., 2013, 2015; Laib Sampaio et al., 2016).

When comparing the gene sequences coding for the subunits of the two complexes, it is striking that solely the gO gene shows a high level of variation among different HCMV strains, clustering into eight distinct gO genotypes (GT). The codon sequence diversity between genotypes ranges from 11 to 24%, but the intra-genotype diversity is less than 2%. The most variable regions are the N-terminus and the central part, and the full length of gO varies between 457 and 472 amino acid residues (Paterson et al., 2002; Rasmussen et al., 2002; Mattick et al., 2004; Stanton et al., 2005). It appears that gO gene-disrupting mutations do not emerge in HCMV strains present in clinical isolates (Sijmons et al., 2015). Furthermore, the different gO genotypes have been found in clinical isolates from diverse geographical locations and in different disease backgrounds, either emerging as a single genotype or a gO genotype mixture (Rasmussen et al., 2002, 2003; Mattick et al., 2004; Stanton et al., 2005; Puchhammer-Stöckl et al., 2006; Bates et al., 2008; Yan et al., 2008; Sowmya and Madhavan, 2009; Görzer et al., 2010, 2015; Chen et al., 2016). This strongly suggests that all distinct gO genotypes are able to fulfill their *in vivo* function given that the integrity of the protein is not disturbed. This is well consistent with the notion that all gO genotypes form stable gH/gL/gO complexes when reconstructed by Ad vector expression (Zhou et al., 2013).

In cell-culture adapted HCMV strains a deletion of gO causes severe reduction in cell-free infectivity on fibroblasts, epithelial, and endothelial cells (Hobom et al., 2000; Dunn et al., 2003; Jiang et al., 2008; Wille et al., 2010), but it has little effect on focal cell-associated spread in the background of HCMV strain Merlin which resembles clinical isolates (Laib Sampaio et al., 2016), indicating that gO plays a particular important role in cell-free infectivity. Since monoclonal anti-gO antibodies are able to neutralize both fibroblast and epithelial cell infection (Gerna et al., 2016; Kabanova et al., 2016), it is suggested that gO is the critical subunit of the trimer, either for interaction with the recently identified fibroblast-specific platelet-derived growth factor alpha receptor (PDGFRα), or for activation of gB-mediated fusion (Schultz et al., 2015; Zhou et al., 2015).

So far, little is known about how the distinct gO genotypes may differ in their function. First evidence came from a study by Paterson et al. (2002) in which they suggested that the sensitivity of focal growth to neutralizing gH antibodies depends on differences in the gO gene. And recently, by analyzing extracellular virions deriving from different HCMV strains, it was proposed that the gO diversity may affect the trimer to pentamer ratio in the virus envelope with potential consequences on HCMV tropism and clinical pathology (Zhou et al., 2013).

In the present study, we aimed to elucidate whether distinct HCMV gO genotypes in an otherwise genetically identical strain background differ in their growth properties. To answer this question we chose two highly diverse gO genotypes for comparison, gO GT1c of HCMV strain TB40E and gO GT4 of strain Towne, which differ by 22% at the amino acid level. First, full-length of gO GT1c of the parental strain TB40-BAC4-luc was exchanged by gO GT4. Second, in these two different gO genotypic backgrounds two strictly conserved cysteine residues were mutated individually and in combination, to generate a set of gO cysteine mutants. Assessment of infection and growth of parental and mutant viruses in fibroblasts and epithelial cells revealed significant differences between GT1c and GT4 in epithelial cell tropism and in the phenotypic manifestation of cysteine point mutations.

## Materials and Methods

### Cells

Human foreskin fibroblasts were cultured in minimum essential medium Eagle (MEM; Sigma–Aldrich, St. Louis, MO, United States) supplemented with 10% heat-inactivated fetal bovine serum (FBS; Gibco Thermo Fisher, Waltham, MA, United States) and 0.5% neomycin (Sigma–Aldrich). Human adult retinal pigmented epithelial cells (ARPE-19; ATCC, Manassas, VA, United States) were cultured in Dulbecco’s modified Eagle medium/nutrient mixture F12 (PAN-Biotech, Aidenbach, Germany) supplemented with 10% FBS and 1% penicillin-streptomycin (Thermo Fisher).

### Viral Mutagenesis

The gO genotype (GT) 4 mutants were generated using the HCMV strain TB40/E as parental strain that was cloned in a bacterial artificial chromosome (BAC) sequence [TB40-BAC4-luc; (Scrivano et al., 2011)]. By “en passant” mutagenesis in *Escherichia coli* GS1783 (Tischer et al., 2010), the glycoprotein O (gO) sequence of the parental strain (gO GT1c) was fully exchanged by gO GT4. For this, a marker cassette was amplified from pEP-Kan-S (kindly provided by N. Osterrieder) by PCR using primer pair 1 (see Supplementary Table 1). The cassette contained a kanamycin resistance gene, flanked on one site by an 18-bp I-Sce I restriction sequence and a gO GT-specific 50-bp sequence, and on both sites by a Sac I restriction site. A plasmid containing the gO GT4 sequence, interrupted by the previously mentioned 50-bp sequence and a Sac I restriction site was ordered from Eurofins Genomics (Luxembourg). The marker cassette and the plasmid were cut with Sac I and ligated. From the resulting transfer plasmid, a recombination cassette was generated by PCR using primer pair 2 (see Supplementary Table 1). The recombination cassette contained extensions of ∼50 bp sequences on each end, which were homologous to the site of insertion in the TB40-BAC-luc genome. The transfer cassette was electroporated into recombination-competent *E. coli* GS1783 carrying the TB40-BAC4-luc genome. For generation of cysteine mutants, single cysteines within gO of GT1c and GT4 were mutated to serine. For this, a recombination cassette was generated directly from pEP-Kan-S as described above. The cassette contained the sequence duplication necessary for red recombination and the gO GT-specific sequence harboring the cysteine mutation, and was electroporated into *E. coli* GS1783 carrying either the TB40-BAC4-luc genome or the TB40-BAC4-luc-gO GT4 genome. Single mutants were further used to generate double cysteine mutants by the described method. After electroporation, recombination-positive *E. coli* were subjected to kanamycin selection, and the introduced non-HCMV sequences were removed within *E. coli* by cleavage at the I-Sce I site and a second red recombination. Positive, i.e., kanamycin-sensitive, bacteria were selected, and overnight cultures were stored at -80°C until further use. Mutant BACs were confirmed by sequencing.

### Viral Reconstitution

Bacterial artificial chromosome-DNA was purified from *E. coli* using the Nucleobond BAC100 kit (Macherey-Nagel, Düren, Germany). The pp71-encoding plasmid pCMV71 (kindly provided by Mark Stinski, University of Iowa) was transformed into NEB10-beta competent *E. coli* (New England Biolabs, Ipswich, MA, United States) by heat-shock, and subsequently purified from overnight cultures using Qiaprep Spin Miniprep kit (Qiagen, Hilden, Germany). For transfection, HFFs were seeded in 6-well plates (3 × 10^5^ cells/well). The following day, 2 μg of BAC DNA and 1 μg of pCMV71 DNA were mixed together with 9 μl of ViaFect reagent (Promega, Madison, WI, United States) and 100 μl of MEM. The mixture was incubated for 15 min at room temperature and then added to the cells, which were incubated further at 37°C. 24 h after transfection, cells were washed with PBS and fresh MEM was added. One week after transfection, cells were trypsinized and transferred into 75 cm^2^ cell culture flasks. Cells were screened regularly by light microscopy for the appearance of cytopathic effect (CPE), and supernatants were tested for HCMV-DNA load by qPCR. When CPE was 90–100%, supernatants were centrifuged at room temperature for 20 min at 4000 × *g*. Of note, CPE of gO GT4-C336S remained restricted to single foci even after HFFs were detached by trypsin and reseeded two times during the course of reconstitution (at days 13 and 18 post transfection) to enhance viral spread. Thus, for gO GT4-C336S virus stocks were harvested when similarly high HCMV-DNA levels were reached. Cleared supernatants, containing virus particles, were stored as viral stocks in aliquots at -80°C, before further usage for infection and replication analyses. All stocks were subjected to whole genome sequencing.

### DNA Extraction

In order to remove non-encapsidated viral DNA and free cellular DNA, viral stocks and cell culture supernatants were treated with Turbo DNase (Thermo Fisher). For this, 100 μl of master mix (73 μl H_2_O, 20 μl 10x DNase buffer, 5 μl 10x PBS, 2 μl Turbo DNase (2 μ/μl)) were added to 100 μl of sample and incubated for 1 h at 37°C in a thermoshaker at 1400 rpm. Thereafter, 2 μl of Protease (100 mg/ml, Qiagen) were added and incubated for 30 min at 37°C in a thermoshaker at 1400 rpm. Viral or cellular DNA was extracted from DNase-treated samples or from non-treated cell suspensions using the bead-based NucliSens EasyMag extractor (BioMérieux, Marcy-l’Étoile, France). DNA was eluted in 50 μl of nuclease-free H_2_O.

### qPCR

For quantification of viral genomes, a region within US17 of the HCMV genome was amplified using the forward primer GCGTGCTTTTTAGCCTCTGCA (10 pM), the reverse primer AAAAGTTTGTGCCCCAACGGTA (10 pM), and the TaqMan probe FAM-TGATCGGCGTTATCGCGTTCTTGATC-TAMRA (2 pM). For quantification of cellular genomic DNA (gDNA), a region within the human beta-2-microglobulin gene was amplified using the forward primer TGAGTATGCCTGCCGTGTGA (3 pM), the reverse primer ACTCATACACAACTTTCAGCAGCTTAC (3 pM), and the TaqMan probe FAM-CCA TGTGACTTTGTCACAGCCCAAGATAGTT-TAMRA (1 pM). Primers and probe (all TIB Molbiol, Berlin, Germany) were mixed with 12.5 μl of TaqMan Universal PCR Master Mix (Applied Biosystems, Foster City, CA, United States) to a volume of 20 μl, which were added to 5 μl of extracted DNA for amplification. PCR amplicons were detected using the ABI 7300 real-time PCR system (Applied Biosystems). The results were compared to an HCMV standard (strain AD169; Advanced Biotechnologies Inc., Eldersburg, MD, United States) and calculated as genome copies/ml. A defined number of human cells was used as standard for calculation of cellular genomic DNA (gDNA).

### Fifty Percent Tissue Culture Infective Dose (TCID_50_) Limiting Dilution Assay

For determination of infectious viral titer, limiting dilution assay was performed. HFFs were seeded in 96-well plates (1 × 10^4^ cells/well) and infected with serial dilutions of viral stock or cell culture supernatant in eight replicates per dilution. 14 days after infection, cells were screened by light microscopy for CPE, and viral titers were calculated as TCID_50_/ml by the Reed–Muench method.

### Infection Efficiency

Human foreskin fibroblasts and ARPE-19 cells were seeded in white, clear, flat-bottom 96-well plates (Corning, Corning, NY, United States) at a density of 1 × 10^4^ cells/well. The following day, viral stocks were treated with DNase as described above, the number of encapsidated genomes of each aliquot was determined by qPCR, and the stocks diluted to the indicated concentrations in cell culture medium; 100 μl of viral dilution per well were used to infect the cells in triplicates for 2 h at 37°C. (MOI of 1 corresponds to 3.59 × 10^8^ encapsidated genomes/ml in gO GT1c). Cells were washed three times with PBS, supplied with 100 μl of medium or medium supplemented with 300 μg/ml of phosphonoacetic acid (PAA; Sigma–Aldrich) and incubated further at 37°C. 48 h post infection, luciferase assay of cell lysates was performed according to the manufacturer’s protocol (Steady-Glo Luciferase Assay System, Promega) and relative light units (RLU) were measured in a Victor Light 1420 plate reader (PerkinElmer, Waltham, MA, United States). Mean RLUs of triplicates were calculated for evaluation.

### Replication Efficiency

**FIGURE 1:**
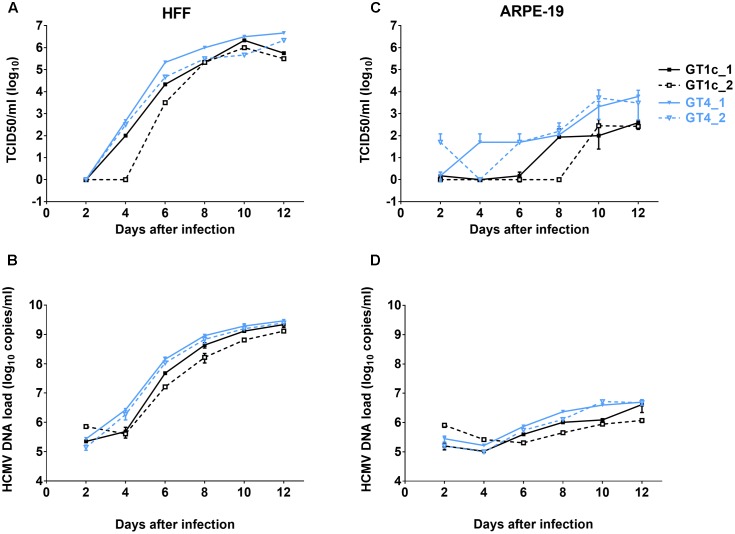
Growth curves and genome dynamics of gO GT1c and gO GT4. Fibroblasts (HFF) and epithelial cells (ARPE-19) were infected with two clones each of gO GT1c and GT4 at an MOI of 0.1 and cultured for 12 days. At the indicated time points, viral titer [TCID_50_/ml on fibroblasts; **(A,C)**] and cell-free viral load [HCMV DNA copies/ml **(B,D)**] of cell culture supernatants were assessed. Error bars indicate SD from three replicates.

One day prior to infection, HFFs and ARPE-19 were seeded in 24-well plates at a density of 1 × 10^5^ cells/well. Viral stocks were diluted in the appropriate cell culture medium. In **Figure 1** cells were infected with an MOI of 0.1. In **Figure 5** initial infectivity was normalized for each strain to yield 500–1500 RLUs at 48 h post infection. Cells were infected with the RLU-adjusted viral dilutions, and 48 h post infection the RLUs were again determined to confirm normalized infectivity of all strains (see Supplementary Table 1). In Supplementary Figure 1 cells were infected with the same number of encapsidated genomes (3.59 × 10^7^/ml) corresponding to an MOI of 0.1 of gO GT1c. In each experiment cells were infected in triplicates for 2 h at 37°C, washed three times with PBS and supplied with 1 ml of medium. Supernatants and cells were harvested separately at indicated time points over a time course of 2 weeks. Viral load and human genomic DNA (gDNA) were determined by qPCR. For cell-associated viral DNA, viral genomes were normalized to the same copy number of human gDNA. Growth curves were generated by assessing viral titers (TCID_50_/ml) of cell-free supernatants at the indicated time points.

### Whole Genome Sequencing

DNA from BAC purification (as described above) and extracted DNA from DNase-treated viral stocks and cell culture supernatants were quantified using the Qubit 2.0 fluorometer (Thermo Fisher) according to the manufacturer’s instructions. One to 2 ng of DNA per sample were taken for library preparation using the Nextera XT DNA Library Preparation Kit and uniquely indexed samples using the Nextera XT Index Kit were pooled and sequenced together. Pooled libraries were sequenced with paired-end reads (2 × 150–250) on a MiSeq system (Illumina, San Diego, CA, United States). Data were analyzed by CLC genomics workbench 8 software (Qiagen). Average >Q30 score ranged between 66 and 92% of reads, with average 867-fold coverage. Low-quality reads were trimmed before alignment to the respective reference sequence.

### Viral Stocks for Immunoblotting

For immunoblotting, supernatants from infected HFFs were harvested when cells displayed ∼90% CPE. Supernatants were precleared from cellular debris by centrifugation for 10 min at 4000 × *g*, before centrifugation for 80 min at 70000 × *g* at 10°C. The virion pellets were resuspended in TAN buffer (0.05 M triethanolamine, 0.1 M NaCl, pH 8) on ice, and stored in aliquots at -80°C.

### Immunoblotting

For sample preparation, virus stocks were mixed undiluted or diluted in TAN buffer with an equal volume of reducing 2x sample buffer (125 mM Tris/Cl pH 6.8, 6% SDS, 10% glycerol, 10% 2-mercaptoethanol, 0.01% bromophenol blue) and incubated on ice for 10 min before boiling at 95°C for 10 min. Samples were separated on 10% SDS PAGE gels together with a high-range rainbow marker (Amersham ECL High-Range Rainbow Molecular Weight Marker, GE Healthcare, United Kingdom). Separated proteins were transferred to polyvinylidene difluoride (PVDF) membranes (Immun-Blot, Bio-Rad, Richmond, CA, United States) in blotting buffer (40 mM Tris, 39 mM glycine, 1.3 mM SDS, 20% methanol), which were than incubated overnight in blocking buffer (PBS, 1% BSA, 0.1% Tween-20) at 4°C. All antibodies were diluted in blocking buffer. Primary mouse anti-major capsid protein (MCP) mAb, anti-gH (AP86-SA4) mAb (both kindly shared by Michael Mach), and anti-gO.02 mAb (kindly provided by Barbara Adler) were incubated for 2 h at RT. Sheep, anti-mouse IgG-HRP (Amersham, GE Healthcare, United Kingdom) was used as secondary antibody and incubated for 1 h at RT. SuperSignal West Femto Maximum Sensitivity substrate (Thermo Fisher) was applied to the membranes according to the manufacturer’s instructions. Chemiluminescent signals were visualized and analyzed using the ChemiDoc Imager and the Image Lab 6.0 software (both Bio-Rad).

### Statistical Analyses

**FIGURE 2:**
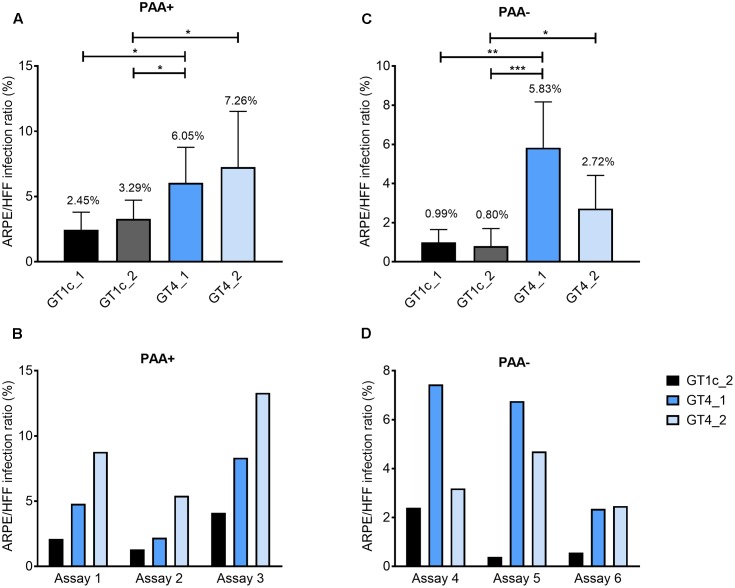
Relative epithelial cell tropism of gO GT1c and gO GT4 mutants. Fibroblasts (HFF) and epithelial cells (ARPE-19) were infected in triplicates with two clones each of gO GT1c and GT4 at low MOIs from 0.1 to 0.4 for 2 h, and incubated for further 46 h in the presence **(A,B)** or absence **(C,D)** of phosphonoacetic acid (PAA). The HFF infection capacity was set to 100% and the epithelial cell infection capacity was determined proportionally. Relative light units (RLU) were determined in triplicates. Mean values from three to six independent experiments are shown in the upper panels, error bars indicate SD. ^∗^*p* < 0.05, ^∗∗^*p* < 0.01, ^∗∗∗^*p* < 0.001, unpaired two-tailed *t*-test. In the lower panels **(B,D)**, three single representative assays for each PAA+ and PAA– are shown.

**FIGURE 3:**
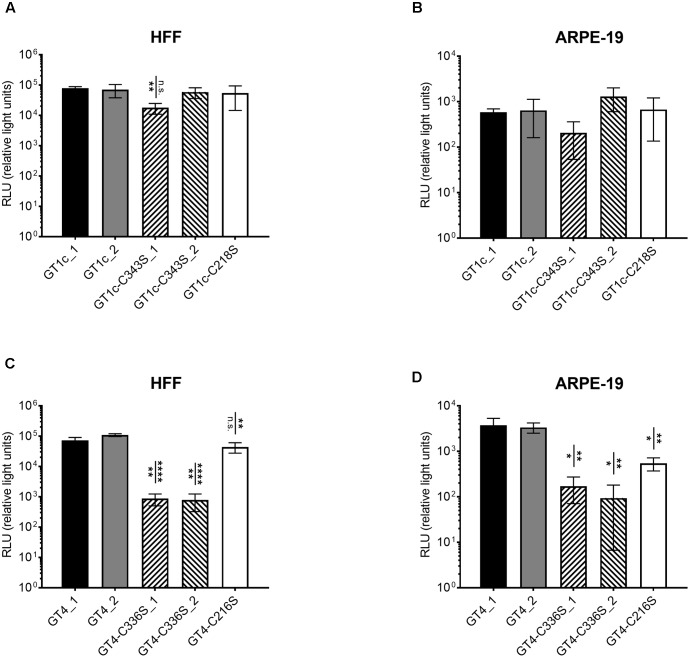
Infectivity of single cysteine mutants for fibroblasts and epithelial cells compared to the respective parental strains gO GT1c and gO GT4. Fibroblasts (HFF) and epithelial cells (ARPE-19) were infected for 2 h with strains of the GT1c-group **(A,B)** or of the GT4-group **(C,D)** with 3.59 × 10^8^ encapsidated genomes/ml, which correspond to an MOI of 1 in GT1c_1. Luciferase assay was performed 48 h post infection and RLUs were determined in triplicates and three independent experiments were performed. Error bars indicate SD. Mean RLUs of the mutants were compared individually to both respective parental clones. Mutants that are significantly different to at least one of the two parental clones are marked by asterisks. ^∗^*p* < 0.05, ^∗∗^*p* < 0.01, ^∗∗∗∗^*p* < 0.0001, unpaired two-tailed *t*-test.

Relative epithelial cell tropism between parental and mutant gO strains (**Figure 2**), and fibroblast and epithelial cell infectivity of cysteine mutants compared to their parental strains (**Figure 3**) were analyzed by unpaired two-tailed *t*-test. To compare infectivity between the two GT1c clones and two GT4 clones (**Figure 3**), differences in mean RLUs were analyzed by one-way ANOVA test. Mean values from three to eight independently repeated experiments were used for statistical analyses. *P*-values < 0.05 were considered significant. GraphPad Prism version 7.01 was used for statistical analyses.

## Results

### Generation of BAC-Derived HCMV Strains Differing Only in the gO Genotype Sequences

To investigate whether the exclusive exchange of the full-length open reading frame (ORF) of gO with a different genotype sequence influences the growth properties of HCMV, the gO GT4 mutant was generated. We used the BAC clone TB40-BAC4-luc as parental strain to seamlessly replace its resident gO GT1c sequence by gO GT4. As previously reported, TB40-BAC4-luc (from now on termed gO GT1c) carries a luciferase expression cassette which allows a sensitive and quantitative measurement of infection over a wide range of multiplicities of infection (MOI) (Scrivano et al., 2011). After “en passant” mutagenesis the genomic integrity of two resulting viral BAC-mutant gO GT4 clones (**Table 1** and Supplementary Table 1) which showed complete replacement of GT1c ORF (464 aa) by GT4 ORF (457 aa) was confirmed by whole genome deep sequencing. Reconstitution of gO GT1c and gO GT4 mutant BAC clones in HFFs resulted in infectious viruses with similarly high TCID_50_ and similar copy numbers of encapsidated viral genomes (**Table 1**). Both, gO GT1c and gO GT4 induced the characteristic HCMV CPE, with even distribution over the entire HFF monolayer. Hence, full-length replacement of gO GT1c by GT4 sequence allowed the generation of infectious virus particles upon reconstitution in HFFs. Whole HCMV genome sequencing of both gO GT1c (GT1c_1 and GT1c_2) and gO GT4 mutant virus stocks (GT4_1 and GT4_2) confirmed that no additional mutations emerged during reconstitution.

**Table 1:** Summary of gO genotype (GT) mutants.

Name of gO mutants	Mutation of gO^1^	CPE during reconstitution in HFFs	TCID_50_/ml (log_10_)	Mean encapsidated genomes/ml (log_10_)^2^	Difference mean encapsidated-TCID_50_ in log_10_ values
GT1c_1	TB40-BAC4-luc	Evenly spread	7.12	9.57	2.45
GT1c_2	TB40-BAC4-luc	Evenly spread	6.60	9.84	3.24
GT4_1	Full-length replacement	Evenly spread	6.57	9.80	3.23
GT4_2	Full-length replacement	Evenly spread	6.57	9.54	2.97
GT1c-C343S_1	Single cysteine to serine	Evenly spread	5.75	9.32	3.57
GT1c-C343S_2	Single cysteine to serine	Evenly spread	6.60	9.66	3.06
GT4-C336S_1	Single cysteine to serine	Clusters of infected cells	4.50	9.60	5.10
GT4-C336S_2	Single cysteine to serine	Clusters of infected cells	4.50	9.65	5.15
GT1c-C218S	Single cysteine to serine	Evenly spread	6.39	9.19	2.80
GT4-C216S	Single cysteine to serine	Evenly spread	5.90	9.39	3.49
GT1c-C218/343S	Double cysteine to serine	Very few single foci	No titer determinable	nd	nd
GT4-C216/336S	Double cysteine to serine	No CPE observed	No titer determinable	nd	nd

^1^*See Supplementary Information for more detailed sequence information.*

^2^*Mean of three quantifications by qPCR of three individual viral stock aliquots.*

*GT, genotype; gO, glycoprotein O; CPE, cytopathic effect; HFFs, human foreskin fibroblasts; TCID_*50*_, Fifty percent tissue culture infective dose; nd, not determined*.

### Multi-Step Growth Curve Analysis of gO GT1c and gO GT4 Mutants in Fibroblasts and Epithelial Cells

TB40E-derived strain TB40-BAC4-luc is able to infect and replicate in a broad range of different cell types including fibroblasts and epithelial cells (Scrivano et al., 2011). To compare the replication efficiencies of the two HFF-derived gO GT4 mutants, GT4_1 and GT4_2, with that of two independently generated HFF-derived gO GT1c virus stocks (GT1c_1 and GT1c_2), HFFs and ARPE-19 cells were infected at an MOI of 0.1. Over a time course, the production of cell-free infectious virus was assayed by limiting dilution assay (TCID_50_) and the amount of HCMV-DNA in supernatant was determined by HCMV-specific qPCR. As shown in **Figure 1**, in HFFs both gO GT4 mutants displayed similar release of cell-free infectious virus and HCMV-DNA load in comparison to the two gO GT1c viruses. Also, the endpoint titers (**Figure 1A**) and endpoint HCMV-DNA levels were similarly high in gO GT1c and gO GT4 strains (**Figure 1B**). In ARPE-19 cells, however, both gO GT4 clones produced 0.89–1.36 log_10_ TCID_50_/ml higher endpoint titers than the gO GT1c clones (**Figure 1C**). In overall, the HCMV-DNA load kinetics in supernatant paralleled the kinetics of cell-free virus production (**Figure 1D**).

These findings demonstrate that despite replacement of the entire resident gO gene sequence of TB40-BAC4-luc by a different gO gene sequence, the resulting viruses retained the growth capacity in different cell types.

### Relative Epithelial Cell Tropism of gO GT1c and gO GT4 Mutants

As gO GT4 displayed a subtle enhancement for growth in ARPE-19 cells compared to GT1c the relative epithelial cell tropism was explored. Therefore, HFFs and ARPE-19 cells were synchronously infected at a low MOI (0.1–0.4) by gO GT1c and gO GT4, and after 48 h cell lysates were assayed for expression of luciferase as indicator for viral infection. A number of independent experiments was performed either with or without the replication inhibitor PAA. In each experiment RLUs were determined in triplicates and the ARPE-19 to HFF infection ratio in percent was determined. As shown in **Figure 2**, each of the two gO GT4 mutant viruses, GT4_1 and GT4_2, showed a higher epithelial cell tropism compared to gO GT1c viruses GT1c_1 and GT1c_2. In the presence of PAA (**Figure 2A**) the epithelial cell tropism was significantly increased for GT4_1 when compared to GT1c_1 (*p* = 0.0292), and for both GT4_1 and GT4_2 when compared to GT1c_2 (*p* = 0.0148 and 0.0169, respectively). Similarly, in the absence of PAA (**Figure 2C**), the ARPE/HFF infection ratio of GT4_1 was significantly enhanced when compared to GT1c_1 (*p* = 0.007), and both of GT4_1 and GT4_2 when compared to GT1c_2 (*p* = 0.0005 and 0.0318), respectively. Similarly as reported by Scrivano et al. (2011), we also observed that the values obtained with this assay are influenced by varying quality of cells and passage number. Nevertheless, when plotting single assays (**Figures 2B,D**), the pattern of enhanced epithelial tropism of both GT4 clones is always maintained.

These data show that replacement of gO GT1c by gO GT4 results in a higher epithelial cell tropism and this most likely leads to the higher release of infectious gO GT4 viruses than gO GT1c viruses when the same MOI is used for epithelial cell infection.

### Generation of a Set of Cysteine to Serine Mutations in gO GT1c and gO GT4 Genotypic Background

Next, to gain more insight into gO genotype-dependent variances we aimed to compare the phenotype of both gO genotypes upon introduction of point mutations. Since mass spectrometry and mutagenesis analysis of the trimeric gH/gL/gO complex recently revealed that the amino acid residues gO-Cys-351 and alternatively gO-Cys-226 (numbering according to gO GT5 of Merlin strain) play important roles in trimer formation and/or stability (Ciferri et al., 2015; Stegmann et al., 2016), we mutated these highly conserved cysteines to serine in gO GT1c (C218S and C343S) and gO GT4 (C216S and C336S), either individually or in combination (**Table 1** and Supplementary Figure 1). During the course of reconstitution in HFFs from all newly generated BAC mutant clones, the CPE was visually inspected by light microscopy and the release of HCMV-DNA in supernatant was measured by qPCR (**Table 1**). The genome sequence correctness of all clones was confirmed before and after reconstitution.

Among all single cysteine mutants only the two gO GT4-C336S clones displayed a CPE that differed from the parental strain-like phenotype (even distribution over the whole monolayer) in that only few scattered clusters of infected cells were seen (**Table 1**). Virus stocks were harvested from the supernatant 27–28 days post transfection when all cysteine mutants reached comparable levels of HCMV-DNA. While also the genome copy numbers of encapsidated HCMV-DNA were similar among all single cysteine mutants, the TCID_50_/ml values which were titrated on HFFs revealed a 2 log_10_ TCID_50_/ml lower virus titer for the two gO GT4-C336S mutant clones compared to parental strain gO GT4 (**Table 1**). Furthermore, the calculated difference in mean log_10_ values between encapsidated genomes and TCID_50_/ml was 5.10 and 5.15 for the GT4-C336S mutants, while for all other strains it ranged from 2.45 to 3.49.

When both cysteine residues, C218 and C343 in GT1c or C216 and C336 in GT4, were mutated, no infectious particles could be detected in supernatant upon reconstitution in HFFs. However, at day 48 post transfection and after several rounds of cell reseeding, the gO GT1c-C218/343S double mutant displayed single foci of infected cells and this phenotype resembled the phenotype of the gO deletion mutant (Jiang et al., 2008; Wille et al., 2010) (**Table 1**). No viral titer was determinable and, hence, no further experiments were performed with the double mutants.

### Relative Infectivity of Single Cysteine Mutants for Fibroblasts and Epithelial Cells in gO GT1c and gO GT4 Background

Since the gO GT4-C336S mutants were severely reduced in HFF-assessed viral titer despite normal amounts of encapsidated genomes when compared to all other viral strains, this is indicative that mutation of C336 in GT4 substantially affects HFF infectivity. Thus, in a next step, we aimed to determine the fibroblast and epithelial cell infection capacities of all cysteine mutant viruses in comparison to their parental strains gO GT1c and gO GT4, respectively. To this end, HFFs and ARPE-19 cells were infected with the same amount of encapsidated virus genomes, corresponding to an MOI of 1 in gO GT1c, to ensure that the same numbers of virus particles were used for the parental and cysteine mutant strains. Infectivity was quantified by monitoring luciferase expression in cell lysates 48 h post infection as mentioned above and three independent experiments were performed. As shown in **Figures 3A,B** and **Table 2**, while both gO GT1c cysteine mutants, gO GT1c-C218S and gO GT1c-C343S, displayed a similar infectivity for HFFs as their parental strains GT1c_1 and GT1c_2, the mutation of C336S in gO GT4 led to reduction in infectivity, which was significant when compared to GT4_1 (*p* = 0.0025) and highly significant when compared to GT4_2 (*p* < 0.0001). The markedly reduced infectivity of gO GT4-C336S compared to parental strain GT4 corresponds well to the above mentioned reduced virus titer despite normal number of encapsidated genomes upon reconstitution in HFFs (**Table 1**).

**Table 2:** Summary of mean epithelial cell tropism.

Name of gO mutants	Mean ARPE-19/HFF infection ratio in %	
	PAA-	PAA+
GT1c_1	0.99	2.45
GT1c_2	0.80	3.29
GT4_1	5.83	6.05
GT4_2	2.72	7.26
GT1c-C343S_1	0.98	nd
GT1c-C343S_2	1.81	nd
GT4-C336S_1	16.44	nd
GT4-C336S_2	6.35	nd
GT1c-C218S	1.08	nd
GT4-C216S	1.72	nd

*ARPE-19, adult retinal pigment epithelial cells; HFF, human foreskin fibroblasts; nd, not determined*.

Also, the relative ARPE-19 cell infectivity was not significantly affected by the single mutations, C218S and C343S, in gO GT1c (**Figure 3C**). Notably, subtle differences might be masked due to the rather high variability among distinct virus stocks of GT1c and GT1c-C343S, respectively. In contrast, in the background of gO GT4, both corresponding single mutants GT4-C336S and GT4-C216S, displayed significantly reduced infectivity (**Figure 3D**) compared to the parental strains gO GT4_1 and GT4_2 with a reduction of 22–23% of mean log_10_ RLU caused by gO GT4-C216S (*p* < 0.005) and an even higher reduction of 37% (GT4-C336S_1) to 44% (GT4-C336S_2) attributed to the single point mutation C336S in GT4 (*p* < 0.005).

As shown in **Table 2**, the resulting ARPE-19/HFF ratios were similar between the GT1c cysteine mutants and the two parental gO GT1c clones. This contrasts to GT4 where the mutation C216S led to a decrease in the ARPE-19/HFF ratio, and C336S even caused an increase in epithelial cell tropism when compared to their respective parental gO GT4 strains. This indicates that despite an overall reduction of infectivity, the gO GT4-C336S mutants retained an enhanced epithelial cell tropism. Taken together, these data demonstrate that the impact of gO cysteine mutations on cell-free infectivity for fibroblasts and epithelial cells significantly differs between the two gO genotypic backgrounds, GT1c and GT4.

Of note, the cell-free infectivity of the GT4 clones compared to the GT1c clones was similar in fibroblasts (**Figures 3A,B**; *p* = 0.17; ANOVA) showing a mean fold increase of 1.2 (range: 0.7–2.3) for GT4_1 and 1.8 (range: 1.0 – 2.6) for GT4_2. The cell-free infection capacity for epithelial cells, however, was significantly different between the GT4 and GT1c clones (*p* = 0.0046; ANOVA) (**Figures 3C,D**). The mean fold increase in epithelial cell infectivity was 7.0 (range: 3.2–12.2) for GT4_1 and 6.3 (range: 4.4–9.1) for GT4_2. These data are well in line with the findings that gO GT4 strains displayed an enhanced epithelial cell tropism as shown in **Figure 2**.

### Content of gO and gH in Virions of gO GT1c- and gO GT4 Mutants

**FIGURE 4:**
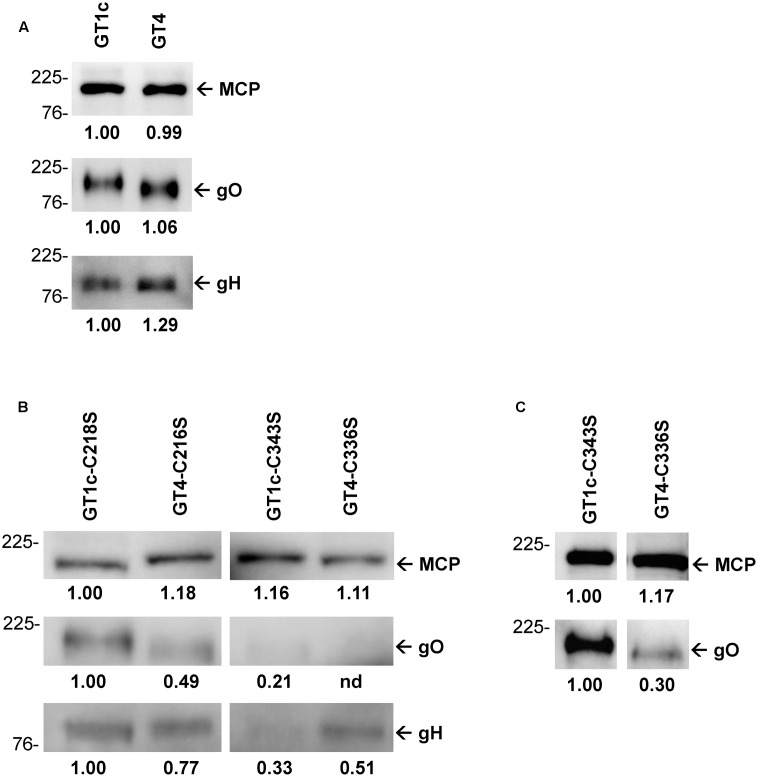
Quantitative Western blots of purified virions. Virions were subjected to reducing gel electrophoresis and analyzed by Western blot using antibodies directed against major capsid protein (MCP), and the glycoproteins gO (anti-gO antibody gO.02) and gH (anti-gH antibody AP86-SA4). The amounts of virions loaded on the gels were normalized to equal amounts of MCP. First, the parental strains GT1c and GT4 were compared with each other **(A)**. Next, the four mutants were MCP-adjusted to each other and compared for content of gO and gH **(B)**. Additionally, the mutants GT1c-C343S and GT4-C336S were analyzed using increased amounts of virions for better detection of gO **(C)**. Band densities were determined relative to one reference band for each blot individually, and are shown below the blots. Mass markers are indicated on the left in kilodalton (kDa).

Glycoprotein O appears to be present in the virions only in a complex with gH/gL and it is suggested that the abundance of gH/gL/gO correlates with the infectivity of the respective virions (Murrell et al., 2013; Zhou et al., 2013, 2015). Thus, we next determined whether the amount of gO and gH in extracellular virions of parental and mutant gO GT1c and GT4 strains differed according to their varying infection capacities as shown above. The gel loads for Western blot analyses under reducing conditions were normalized to MCP which allowed to compare the gO and gH content in the parental und mutant HCMV virions. As shown in **Figure 4A**, the amounts of gO and gH were similar between GT1c and GT4 with a trend toward slightly higher gH in GT4 virions. Quantitative comparison of gO levels among the cysteine mutant virions showed that GT4-C336S contained the lowest amount of gO, followed by GT1c-C343S, GT4-C216S, and GT1c-C218S (**Figures 4B,C**). The severely reduced gO level in GT4-C336S virions corresponds well with the pronounced infection defect as shown above. Consistent with recent findings by Stegmann et al. (2016), the rather low amounts of gO in GT1c-C343S appear to be sufficient to maintain its proper infection capacities for fibroblasts and epithelial cells. Also, the gH contents varied among the gO cysteine mutants, yet the lowest gH levels were observed in GT1c-C343S and not in GT4-C336S virions (**Figure 4B**). These data indicate that mutation of the highly conserved gO cysteine C343 in GT1c or the corresponding C336 in GT4 resulted in a substantial change in the gO and gH content, the extent of reduction, however, depends on the gO genotypic background.

### Replication of gO GT1c-C343S and gO GT4-C336S Mutants in Fibroblasts and Epithelial Cells

**FIGURE 5:**
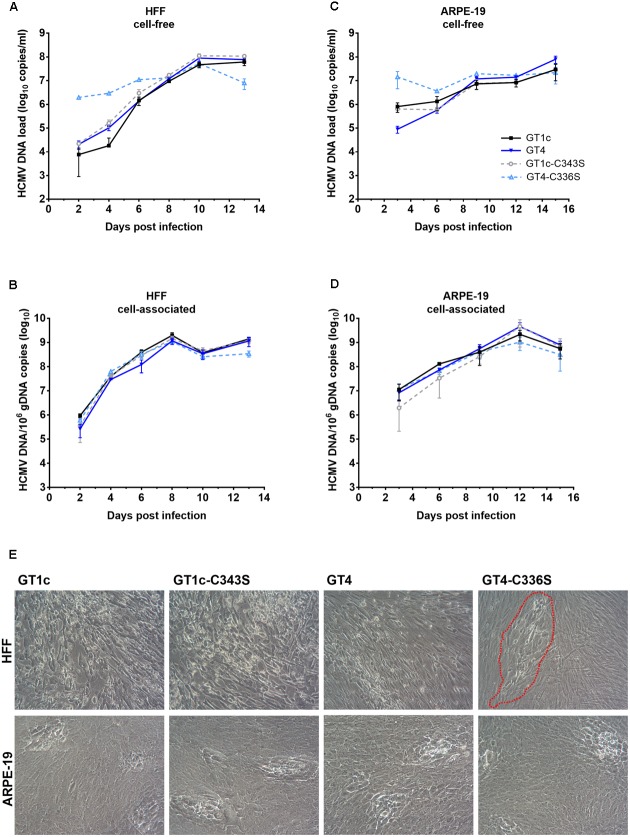
Replication and cytopathic effect of gO GT1c-C343S and gO GT4-C336S mutants. Fibroblasts (HFF) and epithelial cells (ARPE-19) were infected with the mutants GT1c-C34S and GT4-C336S, and their respective parental strains GT1c and GT4 at an MOI normalized to 1000–1500 RLU at 48 h post infection. Infected cells were cultured for 13 (HFF) or 15 (ARPE-19) days. At the indicated time points cell-free **(A,C)** and cell-associated **(B,D)** viral loads were assessed by qPCR. Shown are mean values of three replicates; error bars indicate SD. In **(E)** representative light microscopy pictures of the cytopathic effect of GT1c, GT4 and mutants are shown as seen in HFFs at day 13 and ARPE-19 at day 15 post infection. Infected HFFs of gO GT4-C336S are marked by a red, dashed line.

Finally, we investigated how the cysteine mutations GT1c-C343S and GT4-C336S affect the viral growth behavior in HFFs and ARPE-19 cells in comparison to the corresponding parental strains GT1c and GT4. Since in the previous experiments differences in infectivity among our viral strains were detected (**Figures 2**, **3**), a preliminary dose finding experiment was performed to normalize infectivity of GT1c, GT1c-C343S, GT4, and GT4-C336S to 500–1500 RLUs at 48 h post infection in both cell types individually. This allowed for a matched initial infectivity for the growth curves of all four strains. Then, both cell types were infected with the previously determined virus dilutions (Supplementary Table 2), and cultured for 13 (HFFs) or 15 (ARPE-19) days. The kinetics of cell-free and cell-associated HCMV-DNA were monitored at multiple time-points post infection by qPCR (**Figures 5A–D**).

In HFFs, GT1c, GT1c-C343S, and GT4 displayed similar levels of cell-free and cell-associated HCMV-DNA over the entire time span, and comparable endpoint HCMV-DNA levels (**Figures 5A,B**). The GT4-C336S mutant, on the other hand, showed distinct HCMV-DNA kinetics. In the cell-free fraction, the levels of HCMV-DNA of this mutant were elevated over the first 6 days. This, however, might rather be explained by remnants of the higher amounts of virions used for initial infection (Supplementary Table 2) than by a new production of cell-free HCMV-DNA, since no differences were observed when equal amounts of encapsidated virions were used for initial infection (see Supplementary Figure 1). Also, cell-associated HCMV-DNA kinetics of GT4-C336S until day 10 were rather similar to that of the other strains, independently of whether same amounts of encapsidated virions or normalized infectivity was used for initial infection, which further corroborates that the replication efficiency in HFFs is comparable among all strains.

However, the GT4-C336S mutant showed substantially reduced endpoint HCMV-DNA levels, both in the cell-free (1.0 log_10_ copies/ml difference) and in the cell-associated (0.6 log_10_ copies/ml difference) fraction (**Figures 5A,B**). Since only the initial infectivity could be normalized, it is most likely that in subsequent rounds of infection the reduced fibroblast infection capacity of GT4-C336S virions caused reduced endpoint HCMV-DNA levels. Also, the CPE at day 13 post infection was clearly distinct in that the infected cells remained restricted to single foci (**Figure 5E**). All other strains displayed the characteristic CPE with swollen cells and cell syncytia that were spread over the entire cell monolayer.

In ARPE-19 cells, cell-free HCMV-DNA loads in the early phase post infection were again not uniform between the viral strains due to variety in applied virus amounts (**Figure 5C** and Supplementary Table 2). Of note, no differences in early viral loads were seen when the initial infection was performed with equal amounts of encapsidated virions (Supplementary Figure 1). At day 9 and later, similar amounts of cell-free HCMV-DNA were detected for all strains. GT1c, GT1c-C343S, and GT4-C336S yielded comparable endpoint DNA loads, while GT4 was slightly elevated, corresponding to the result seen in **Figure 2**. Further, no differences between the strains could be observed in the cell-associated HCMV-DNA kinetics (**Figure 5D**) indicating similar replication dynamics with all four virus strains. Further, all four strains induced a similar CPE at day 15 post infection (**Figure 5E**).

Taken together, these data indicate that the marked impairment in cell-free fibroblast infectivity of the gO GT4-C336S mutant leads to an attenuated growth phenotype with lower endpoint HCMV-DNA levels and the observed CPE pattern. On the other hand, the retained epithelial cell tropism (**Table 2**) allows the GT4-C336S mutant to grow in a similar fashion as its parental strain GT4, indicating that the cell-to-cell spread is not affected. The corresponding cysteine mutation in the background of gO GT1c, yet shows a GT1c-like growth phenotype in both cell types. This further underlines that these single cysteine mutations in gO GT1c do not substantially impact the function of gO despite the substantial reduction of the gO content in GT1c-C343S virions as shown above.

## Discussion

In the present study, we show that two identical HCMV strains that differ solely in the gO genotype sequence, either gO GT1c or gO GT4, display significant differences in epithelial cell tropism. We further demonstrate that single point mutations of two strictly conserved cysteines of gO, known to be important for gH/gL/gO trimer formation and/or stability, differentially impair cell-free infectivity for fibroblasts and epithelial cells depending on the gO genotype background in which they were mutated.

First, we could show that the TB40-BAC4-luc-derived mutant virions with full-length replacement of gO GT1c by GT4 ORF sequence of Towne strain (gO GT4 mutants) retained the ability to infect both fibroblasts and epithelial cells. This suggests that the basic functional nature of gO is similar between distinct gO genotypes and this is well in concordance with the findings that all gO genotypes can form stable gH/gL/gO trimers and that different HCMV strains, each of which carrying a distinct gO genotype, also contain the trimeric complex in their virions (Zhou et al., 2013).

**FIGURE 6:**
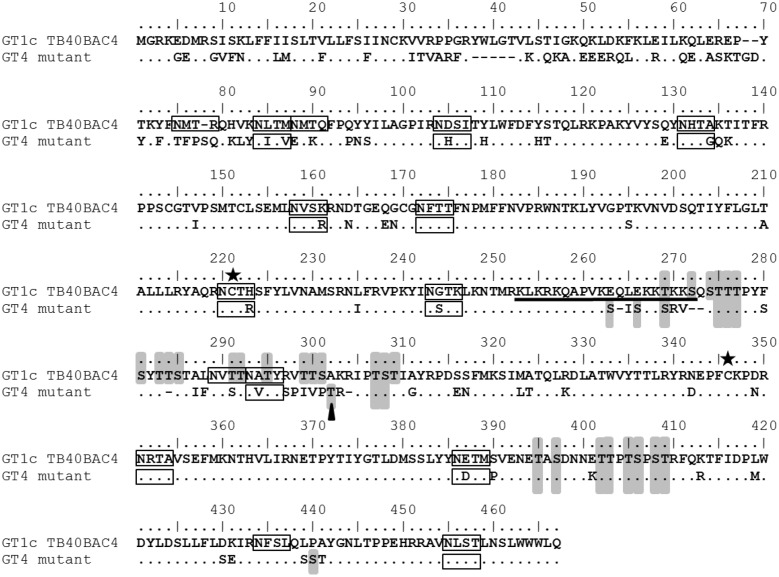
Comparison of gO GT1c and gO GT4 amino acid sequences. Reference sequences of genotype (GT) 1c (TB40BAC4; Protein ID: ABV71596.1) is aligned with gO GT4 sequence of HCMV strain Towne (Protein ID: KF493877.1). Putative *N*-glycosylation sites as predicted by NetNGlyc 1.0 are indicated by black boxes and putative *O*-glycosylation sites as predicted by NetOGlyc 4.0 are highlighted in gray. The black bar indicates the hydrophilic stretch as characterized previously (Zhou et al., 2013). The asterisks show the cysteine residues, GT1c-C218 or GT4-C216, and GT1c-C343 or GT4-C336. The black triangle depicts the *O*-glycosylation site identified by mass spectrometry (Bagdonaite et al., 2016).

Detailed comparison of the infection behavior of gO GT4 mutant viruses compared to parental strain TB40-BAC4-luc (gO GT1c viruses) revealed that gO GT4 mutants displayed a significantly higher epithelial cell tropism than gO GT1c viruses. Despite a rather high variation in the epithelial cell to fibroblast infection ratio between experiments, this phenotype was always seen in a set of 3–6 independent experiments and this was proven for two independently generated gO GT4 mutant virus clones. Besides, similar results were obtained in the presence and absence of the replication inhibitor PAA indicating that the observed differences in epithelial cell infectivity were not due to potential differences in replication. This finding was further supported by the observation that gO GT4 viruses showed a more than sixfold increase in their capacity to infect epithelial cells compared to gO GT1c when from both, gO GT1c and gO GT4 viruses, the same number of encapsidated HCMV DNA genomes was used for infection. Furthermore, we confirmed by whole genome sequencing that no other mutations emerged during the course of reconstitution. Thus, it is strongly suggested that the observed differences in epithelial cell infectivity are attributed to sequence differences between gO GT1c and gO GT4 (**Figure 6**). Notably, of all gO GT sequences, GT4 is the most diverse to GT1c, sharing only 78% identity in codon sequence, and therefore was chosen for gO GT replacement. The GT1c and GT4 sequences differ most at the N-terminus and in the central part of gO, and further inspection for predicted *N*- and *O*-glycosylation sites using the web-based programs NetNGlyc 1.0 and NetOGlyc 4.0, respectively, revealed a substantial difference in the number of predicted *N*-glycosylation (15 sites in GT1c vs. 11 in GT4) and *O*-glycosylation sites (27 in GT1c vs. 18 in GT4) between the two genotypes. Most of the putative *O*-glycan sites are found in the central part of gO (**Figure 6**) which also includes a prominent hydrophilic stretch that is less hydrophilic in GT4 than in GT1c (Paterson et al., 2002; Mattick et al., 2004; Zhou et al., 2013). Hence, it is very likely that the specific sequence properties of GT1c and GT4 may differentially influence the function of gO.

In virions, gO is found in the envelope in a complex with the heterodimer gH/gL (Huber and Compton, 1998). Recent studies have revealed that gH/gL-C144 forms a covalent bond either with gO or UL128 (Ciferri et al., 2015). The resulting trimer to pentamer ratio of the virions appears to influence the cell tropism, whereby the relative levels of the trimer and pentamer can differ between distinct strains, influenced by the expression levels of UL128-131 (Murrell et al., 2013; Zhou et al., 2013, 2015), the viral regulator UL148 (Li et al., 2015), the cell type producing the virion population (Scrivano et al., 2011), and probably also by the gO genotype of the respective strain (Zhou et al., 2013). TB40E-derived strain TB40-BAC4-luc contains vastly more trimer than pentamer due to a UL128 intron mutation which as a consequence displays a cell-free infection capacity that is low for epithelial cells and high for fibroblasts (Murrell et al., 2013). Thus, firstly it is presumable that replacement of TB40-BAC4-luc resident gO GT1c by GT4 caused a switch toward more pentamer and this could well explain the observed increase in epithelial cell tropism, yet by retaining enough trimer for efficient fibroblast infection. Our data, however, show that both strains harbor comparable gO levels in their extracellular virions which strongly suggests that the amount of trimer did not differ between gO GT1c and gO GT4. From our data it appears that the gO GT4 virions contain slightly more gH than gO GT1c virions. If this indicates that gO GT4 harbors more of the gH-containing pentamer complex than gO GT1c yet awaits further clarification. Secondly, it cannot be excluded that genotype-specific sequence properties differentially affect a gO function that is played out by endocytosis-mediated entry into epithelial cells only and not by fibroblast entry (Zhou et al., 2015; Kabanova et al., 2016). Further investigations will be necessary to clarify which parts of the heterogeneous gO sequences contribute to functional differences and how these properties might influence the formation and/or stabilization of the trimer.

Interestingly, the enhanced epithelial cell infectivity of gO GT4 viruses resulted in a slightly higher release of cell-free viruses compared to GT1c as shown by growth curve analysis on epithelial cells. Since HCMV transmission typically occurs via a mucosal route, epithelial cells are supposed to be the first target cell type of HCMV infection and these, after viral dissemination to other cell types, contribute to viral shedding into body fluids (Mocarski, 2013). Thus, it is tempting to speculate that the production of more virions by GT4 in combination with an enhanced cell-free infectivity might have clinical consequences either in viral shedding and/or in the ability of the virions for transmission to another host. However, it has to be considered that all known gO genotypes can be found in clinical samples, yet the frequency of occurrence may differ among distinct gO genotypes (Rasmussen et al., 2003; Mattick et al., 2004; Stanton et al., 2005; Puchhammer-Stöckl et al., 2006; Yan et al., 2008; Görzer et al., 2010, 2015; Chen et al., 2016). Moreover, it is assumed that numerous different HCMV strains circulate in the human population, each characterized by a specific combination of genotypes when all polymorphic genome regions are taken into account (Puchhammer-Stöckl and Görzer, 2011; Sijmons et al., 2015). Thus, it is likely that the infection behavior of an HCMV strain does not solely rely on the functional properties of a unique genotype but rather on a certain genotype combination. However, specific functional features of an individual genotype may either be masked or enhanced depending on its overall genotype-combination and this needs to be explored in further investigations.

Recent mass spectrometry and mutagenesis analysis of HCMV gH/gL/gO revealed that gL-C144 forms a disulfide bond with gO GT5-C351 and may alternatively form a disulfide bond with gO GT5-C226 (Ciferri et al., 2015). In the present study, we also focused on these two strictly conserved gO cysteines and we compared how a mutation to serine affects infection and growth on epithelial cells and fibroblasts either in gO GT1c (C218S and C343S) or in gO GT4 (C216S and C336S) in an otherwise identical HCMV TB40-BAC4-luc genomic background. Our data show that none of the two single cysteine mutations in gO GT1c background led to an explicit change in cell-free infectivity and this is well in agreement with the findings recently published by Stegmann et al. (2016). Remarkably, this is in sharp contrast to gO GT4 in which both of the corresponding cysteine mutations (C216S or C336S) provoked a reduction in cell-free infectivity. Particularly, the strong difference between the gO GT1c-C343S and gO GT4-C336S infection phenotypes was further underlined by their divergent growth curve kinetics on fibroblasts and by their markedly different CPE. We could further show that the mutation of C336 in GT4 causes a more severe reduction in the virion’s gO content than the corresponding C343 mutation in GT1c. And this is well in line with the different infection capacities of these two cysteine mutants. Since, the gO cysteine residues are strictly conserved among the gO genotypes, it is very likely that specific sequence properties of gO GT1c and GT4 as described above differentially influence the compensation of a cysteine loss. Collectively, these findings provide strong indirect evidence that gO GT1c and GT4 differ in their functional behavior.

An interesting finding was that gO GT4-C216S caused only a moderate reduction in infectivity which was played out mainly on epithelial cells whereas the mutation of GT4-C336S led to about 40% impairment in cell-free infectivity for both cell types. This indicates that C336 plays a much more prominent role in maintaining the proper function of gO GT4 than C216 and this is clearly reflected by the much smaller amount of gO in the extracellular virions of GT4-C336S compared to GT4-C216S. Interestingly, the gH content only slightly differs between these two cysteine mutants. Thus, the relatively high gH content in the gO GT4-C336S virions might reflect more pentamer in the virions and this would well explain why gO GT4-C336S displayed a substantial increase in epithelial to fibroblast infection ratio despite an overall impairment in cell-free infectivity for both cell types.

Finally, we could also show that simultaneous mutation of both cysteines to serine almost completely abolished viral growth in the two distinct gO genotypic backgrounds. This indicates that for both gO GT1c and gO GT4 at least one of these highly conserved cysteines has to be present. The phenotype of the double mutants resembled that of the gO deletion mutant (Jiang et al., 2008; Wille et al., 2010; Scrivano et al., 2011; Laib Sampaio et al., 2016; Stegmann et al., 2016), and this is most likely due to disruption of the trimer as recently reported (Stegmann et al., 2016).

## Conclusion

The phenotypic characterization of a set of gO mutants demonstrates that gO GT1c and gO GT4 differ in their functional properties which seem to influence HCMV epithelial cell tropism and this may have important implications on virus host transmission.

## Author Contributions

JK executed experiments. IG and JK designed the experiments. IG and EP-S supervised the experimental work. JK and IG performed data analyses and drafted the manuscript. BA contributed intellectually and provided the HCMV strain TB40-BAC4-luc and the anti-gO antibody. MM and BK kindly provided the anti-gH and anti-MCP antibodies and very useful information for immunoblotting. All authors edited and approved the manuscript.

## Conflict of Interest Statement

The authors declare that the research was conducted in the absence of any commercial or financial relationships that could be construed as a potential conflict of interest.
