# Supplementary material for: Differences in Growth Properties among Two Human Cytomegalovirus Glycoprotein O Genotypes
Source: Front Microbiol. 2017 Aug 22;8:1609. doi: 10.3389/fmicb.2017.01609 (PMC5572245; doi:10.3389/fmicb.2017.01609)
Supplement: Supplementary file 1 [file Data_Sheet_1.DOCX]

***Supplementary Material***

**Differences in Growth Properties among two Human Cytomegalovirus Glycoprotein O Genotypes**

**Kalser Julia, Adler Barbara,** **Mach Michael, Kropff Barbara, Puchhammer-Stöckl Elisabeth, Görzer Irene^*^**

***Correspondence:**

Irene Görzer

irene.goerzer@meduniwien.ac.at

**Supplementary Table 1. Primer for mutagenesis**

| **gO mutant** | **Primer** | **Forward (5’ – 3’)** | **Reverse (5’ – 3’)** |
| --- | --- | --- | --- |
| GT4 | Pair 1 | TAAGGAGCTCATGTCAAGAGTACCGTAAATAGTGTACGGTGTTTCGTTGCGAATCTAGGGATAACAGGGTAATCGATTT | TAGCGAGCTCGCCAGTGTTACAACCAATTAACC |
|  | Pair 2 | CTGCTTTCAGAACTTTACTGCAACCACCACCAAAGGCTATTGAGGGTGGACAGGTTCACAGCCCGGCG | CACAAGGCAGACGGACGGTGCGGGGTTTCCTCCTCTGTCATGGGGAGAAAGGGAGAGATGAGAGGTGTTT |
| GT1c-C343S |  | TAAATTCTGACACGGCGGTACGGTTACGGTCTGGTTTCGAAAAGGGTTCATTCCGATACCTAGGGATAACAGGGTAATCGATTT | ATGGGTGTATACTACTCTGCGGTATCGGAATGAACCCTTTTCGAAACCAGACCGTAACCGGCCAGTGTTACAACCAATTAACC |
| GT4-C336S |  | TAAATTCTGATACGGCGGTCCGGTTACGGTTTGGTTTCGAAAAAGGTTCATCTCGATAGCTAGGGATAACAGGGTAATCGATTT | GTGGGTGTATACTACTCTGCGCTATCGAGATGAACCTTTTTCGAAACCAAACCGTAACCGGCCAGTGTTACAACCAATTAACC |
| GT1c-C218S |  | TCCGGCTCATGGCGTTAACCAGGTAGAAACTGTGTGTACTGTTGCGTTGTGCGTAACGTATAGGGATAACAGGGTAATCGATTT | GGGTTTAACCGCCCTACTTCTACGTTACGCACAACGCAACAGTACACACAGTTTCTACCTGCCAGTGTTACAACCAATTAACC |
| GT4-C216S |  | TCCGGCTCATGGCGTTAACCAGGTAGAAGCTGCGTGTCGAGTTACGTTGCGCGTAACGTATAGGGATAACAGGGTAATCGATTT | AGGTTTAGCCGCCCTGCTTCTACGTTACGCGCAACGTAACTCGACACGCAGCTTCTACCTGCCAGTGTTACAACCAATTAACC |
| GT1cdoubleCS |  | Same as for single mutants | Same as for single mutants |
| GT4doubleCS |  | Same as for single mutants | Same as for single mutants |

**Supplementary Table 2. RLU-normalization of infectivity.** For analysis of replication of gO GT1c-C343S and gO GT4-C336S mutants in fibroblasts and epithelial cells, initial infectivities of all strains were normalized to yield 500-1500 RLUs in each cell type. Indicated are the amounts of encapsidated (encap) viral genomes/ml used for infection, and mean RLUs of triplicates measured 48h post infection for confirmation of the normalized infectivity.

| Virus | HFF encap genomes/ml | HFF RLU 48h pi | ARPE encap genomes/ml | ARPE RLU 48h pi |
| --- | --- | --- | --- | --- |
| GT1c | 8,65E+06 | 1513 | 3,46E+08 | 1045 |
| GT1c-C343S | 1,43E+07 | 742 | 9,54E+07 | 932 |
| GT4 | 7,90E+06 | 545 | 5,27E+07 | 764 |
| GT4-C336S | 3,98E+08 | 901 | 9,95E+08 | 1756 |

**Supplementary Figure 1. Replication and cytopathic effect of gO GT1c-C343S and gO GT4-C336S mutants.** Fibroblasts (HFF) and epithelial cells (ARPE-19) were infected with two independent virus stocks each of GT1c, GT1c-C34S, and GT4-C336S with 3.59 x 10^7^ encapsidated genomes/ml, which corresponds to an MOI of 0.1 in GT1c_1. GT1c_1 was additionally used at an MOI of 0.01, representative of reduced initial infectivity. Infected cells were cultured for 13 (HFF) or 15 (ARPE-19) days. At the indicated time points, cell-free **(A, C)**, and cell-associated **(B, D)** viral loads, were assessed by qPCR. Shown are mean values of 3 replicates; error bars indicate SD.
